# The renal compartment: a hydraulic view

**DOI:** 10.1186/s40635-014-0026-x

**Published:** 2014-10-23

**Authors:** Pablo Cruces, Camila Salas, Pablo Lillo, Tatiana Salomon, Felipe Lillo, Daniel E Hurtado

**Affiliations:** Centro de Investigación de Medicina Veterinaria, Escuela de Medicina Veterinaria, Facultad de Ecología y Recursos Naturales, Universidad Andres Bello, Av. República 237, Santiago, Chile; Pediatric ICU, Hospital El Carmen de Maipú, Santiago, Chile; Pediatric ICU, Clínica Alemana de Santiago, Santiago, Chile; Department of Structural and Geotechnical Engineering and Biomedical Engineering Group, Pontificia Universidad Católica de Chile, Santiago, Chile

**Keywords:** Compartment syndrome, Renal capsule, Intrarenal pressure, Kidney biomechanics

## Abstract

**Background:**

The hydraulic behavior of the renal compartment is poorly understood. In particular, the role of the renal capsule on the intrarenal pressure has not been thoroughly addressed to date. We hypothesized that pressure and volume in the renal compartment are not linearly related, similar to other body compartments.

**Methods:**

The pressure-volume curve of the renal compartment was obtained by injecting fluid into the renal pelvis and recording the rise in intrarenal pressure in six anesthetized and mechanically ventilated piglets, using a catheter Camino 4B® inserted into the renal parenchyma.

**Results:**

In healthy kidneys, pressure has a highly nonlinear dependence on the injected volume, as revealed by an exponential fit to the data (*R*^2^ = 0.92). On the contrary, a linear relation between pressure and volume is observed in decapsulated kidneys. We propose a biomechanical model for the renal capsule that is able to explain the nonlinear pressure-volume dependence for moderate volume increases.

**Conclusions:**

We have presented experimental evidence and a theoretical model that supports the existence of a renal compartment. The mechanical role of the renal capsule investigated in this work may have important implications in elucidating the role of decompressive capsulotomy in reducing the intrarenal pressure in acutely injured kidneys.

## Background

Compartment syndrome is the final result of a process that begins with the persistent increase in pressure within a tissue or parenchyma to such an extent that it is capable of altering the regional vascular inflow. Compartment syndrome may culminate in local organ failure, and if the damage persists, multiple organ failure may occur, resulting in the death of the patient. This is true for any body compartment surrounded by a rigid or semi-rigid structure, a situation often found in intracranial hypertension syndrome or abdominal compartment syndrome [1,2]. From a hydraulic point of view, the ‘renal compartment’, whose content and structure are the parenchyma and renal capsule, respectively, should not be different from other body compartments.

In analogy to the intracranial hypertension syndrome, a sudden increase in fluid volume inside the renal parenchyma can result in a substantial intrarenal pressure and, as a consequence, a decrease in the renal perfusion pressure [3]. In effect, acutely injured kidneys where edema is present typically involve ischemic regions of the outer medulla due to a reduction in the vascular inflow [4,5]. From a clinical perspective, the venous congestion due to an increase of the vascular permeability to proteins, as well as the decrease of the intrarenal perfusion pressure increase the risk of developing a new or persistent septic acute kidney injury (AKI) [6].

The present study aims to understand, both from experimental and biomechanical approaches, the functional dependence between the renal compartment volume increments and changes in the intrarenal pressure mediated by the renal capsule. We hypothesize that pressure and volume in the renal compartment are not linearly related, and may present a functional dependence similar to those found in other compartmental syndromes.

## Methods

### Animal preparation

This study used anesthetized domestic large white piglets purchased from a local vivarium specialized in this species. The Universidad Andrés Bello Ethics Committee approved the experimental protocol. All experimental procedures were in accordance with the Guiding Principles in the Care and Use of Laboratory Animals adopted by the American Physiological Society. The study was powered to detect an increase in intrarenal pressure. Sample size needed to achieve an 80% study power was six, with a 0.05 one-sided significance level and a standard deviation of 33% [7].

#### Surgical preparation and anesthesia

Animals were premedicated with intramuscular midazolam (0.5 mg/kg), methadone (0.5 mg/kg), and ketamine (15 mg/kg), followed by induction with intravenous propofol (3 mg/kg). Tracheal intubation was performed with a cuffed tracheal tube (5.0-mm internal diameter; Mallinckrodt Shiley, St. Louis, MO, USA) for inhalation anesthesia with isoflurane 1.5%. An adequate level of anesthesia is assumed if reflexes are absent. Anesthesia and neuromuscular blockade were maintained by continuous infusion of propofol (10 mg/kg/h), fentanyl (5 μg/kg/h), and vecuronium (0.3 mg/kg/h) throughout the all experiments which lasted for less than 1 h. Heart rate, mean arterial pressure, and temperature were continuously monitored during the whole duration of the experiment. Before laparotomy, PaO_2_, pH, PaCO_2_, serum creatinine, and hemoglobin were assessed with an i-STAT® (Abbott Laboratories, Princeton, NJ, USA) in blood samples from the arterial catheter.

#### Mechanical ventilation

Animals were ventilated with anesthesia workstation Fabius GS® premium (Dräger Medical, Lübeck, Germany) using the volume control mode. Initial settings were: *V*_*T*_ = 10 mL/kg, PEEP = 5 cmH_2_O, fraction of inspired oxygen = 0.4, inspiratory time = 1.0 s, and respiratory rate (RR) = 20 breaths/min. RR was adjusted to achieve a partial pressure of carbon dioxide (PaCO_2_) 40 ± 10 Torr.

#### Pressure-volume curve protocol

**Figure 1:**
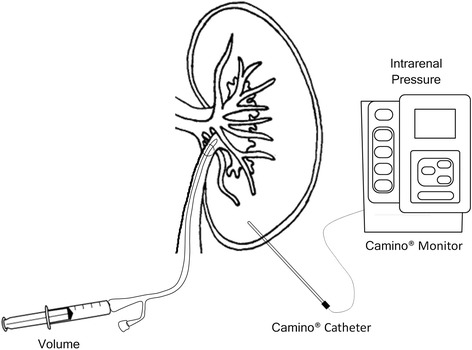
**Schematic of the experimental setup design for measuring the intrarenal pressure.** Fluid is injected through a Foley catheter into the renal pelvis, and intrarenal pressure is measured using a Camino catheter located in the lower renal pole.

After surgical preparation, each animal was placed in supine decubitus and a midline laparotomy was performed. Left kidneys were identified and dissected free of surrounding tissue. Since the kidney can be considered as relatively non-compressive and predominantly fluid in character, subject to Pascal's law, the intrarenal pressure can be measured in nearly every part of the kidney. Intrarenal pressure was measured using a catheter Camino 4B® inserted 1 cm in the left lower renal pole and connected to Camino Single Parameter Monitor Model SPM1 (Integra NeuroSciences, New Jersey, USA). Ureters were sectioned and a 10-Fr Foley catheter into the renal pelvis through the ureter (Figure 1). The abdomen was closed temporarily to maintain thermoregulation. The volume-pressure curve for each animal was obtained by first removing the urine inside the renal pelvis, after which a controlled volume of normal saline was injected and the peak value of the time course of pressure was recorded, to finally draw all the fluid inside the pelvis. This cycle was repeated in time intervals of approximately 3 min. The total injected fluid volume was increased by 1 mL for each subsequent cycle, starting with an injected volume of 1 mL. Following the rapid rise in pressure, the renal pressure was permitted to return to the initial control level before the next injection sequence. In the case of decapsulated kidneys, we followed the decapsulation technique described in [8], where the capsule is first incised and elevated at its lateral margin, then cut from the superior to the inferior pole and finally stripped apart in the medial plane.

While under anesthesia, the animals were euthanized by 10% potassium chloride infusion until the detection of ventricular fibrillation or asystole.

### Statistical analysis

Data are expressed as mean values ± SEM. Normality was assessed with the Anderson-Darling test. The Wilcoxon signed-rank test and the Friedman test with Bonferroni correction were conducted to compare consecutive measurements of studied variables. Significance was set at *P* < 0.05. All statistical analyses were performed using SPSS 20.0 (SPSS Inc., Chicago, IL, USA).

## Results

Six anesthetized and mechanically ventilated piglets were studied (12.3 ± 2.4 kg). The baseline characteristics are described in Table 1 . All of the animals completed the experimental protocol.

**Table 1:** **Baseline characteristics of the piglets included in the study**

	**Baseline**
HR (bpm)	97 ± 11
MAP (Torr)	85.5 ± 5.7
Temperature (°C)	37.6 ± 0.2
pH	7.43 ± 0.03
PaO_2_ (Torr)	171.2 ± 6.2
PaCO_2_ (Torr)	43.1 ± 3.1
Serum creatinine (mg/dL)	1.13 ± 0.05
Hemoglobin (g/dL)	9.0 ± 0.3

Data is expressed as mean value ± SEM. HR, heart rate; MAP, mean arterial pressure.

**Figure 2:**
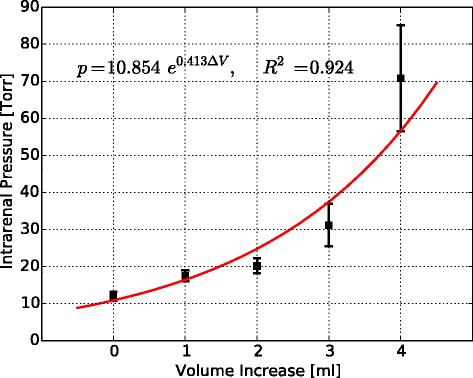
**Pressure-volume curve in the renal compartment.** Data is expressed as mean value ± SEM. *Solid line* represents the exponential fit to the data. p, intrarenal pressure; V, volume injected to the renal pelvis.

The pressure-volume experimental data are shown in Figure 2 and summarized in Table 2. The measured intrarenal pressure increased progressively as successive volume increments of normal saline were injected into the renal pelvis. The pressure-volume curve in normal kidneys exhibited a marked nonlinear behavior. In particular, it can be observed from Figure 2 that the pressure is not proportional to the volume injected. An exponential expression was found to give the best fit to the data, resulting in the following mathematical relation:

**Table 2:** **Pressure-volume data in the renal compartment**

	***V*** **(mL)**				
	**0**	**1**	**2**	**3**	**4**
Intrarenal pressure (Torr)	12.0 ± 2.1	17.5 ± 2.9	20.2 ± 3.4	31.2 ± 6.8	70.8 ± 16.7

Data is expressed as mean value ± SEM. V, volume injected to the renal pelvis.

$$ p=10.854\ {e}^{0.413\ \varDelta V}\kern0.5em \left[\mathrm{Torr}\right] $$

where *p* is the intrarenal pressure and *ΔV* is the volume of the normal saline injected to the renal pelvis.

### Biomechanical model

**Figure 3:**
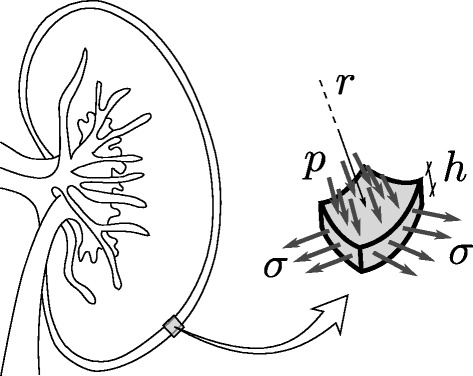
**Schematic of the biomechanical model for the kidney capsule.** p, intrarenal pressure; σ, capsule stress; h, capsule thickness; r, capsule radius.

We model a portion of the renal capsule as a membrane with spherical-cap shape subject to an internal pressure exerted by the renal parenchyma acting as a fluid (Figure 3). Let *p* be the fluid pressure, *σ* be the capsule stress (force per unit area), and *h* and *r* be the capsule thickness and the radius of the capsule in the deformed state, respectively. From Laplace's law, the intrarenal pressure and capsule stress are related through

1 $$ p=2\frac{h}{r}\ \sigma $$

The capsule stress is related to its deformation through a constitutive law. Nedeker et al. [9] performed extensive mechanical testing of porcine kidney capsules, proposing an incompressible hyperelastic Ogden model [10]. For a capsule element under equi-biaxial loading subject to internal pressure [11], the constitutive law reads

2 $$ \sigma ={\mu}_1\ \left({\lambda}^{15}-{\lambda}^{-30}\right)+{\mu}_2\ \left({\lambda}^{7.5}-{\lambda}^{-30}\right) $$

where *μ*_1_ = 0.0015 Torr (0.2 MPa) and *μ*_2_ = 0.0315 Torr (4.2 MPa) are material constants determined from experiments [6], and *λ* is the stretch ratio, defined as the ratio between the length of a segment after and before deformation. Let *H* and *R* be the undeformed capsule thickness and radius, respectively. The incompressibility of the tissue implies

3 $$ \frac{h}{r}=\frac{H}{R}\times \frac{1}{\lambda^3} $$

The increase in volume Δ*V* due to fluid injection relates to the stretch by

4 $$ \Delta V=\left({\left({\lambda}_{\Delta}\right)}^3-1\right){V}_0 $$

where *λ*_Δ_ is the incremental stretch ratio. Let *λ*_0_ be the stretch ratio of the capsule before the fluid injection. Then, the total capsule stretch is

5 $$ \lambda ={\lambda}_0 \times {\lambda}_{\Delta} $$

By combining Equations 1 to 5, we obtain the following nonlinear expression for the renal pressure as a function of the injected fluid volume

6 $$ p=2\frac{H}{R}\left[{\mu}_1\left\{{\lambda}_0^{12}{\left(1+\frac{\Delta v}{V_0}\right)}^4-{\lambda}_0^{-33}{\left(1+\frac{\Delta v}{V_0}\right)}^{-11}\right\}+{\mu}_2\left\{{\lambda}_0^{4.5}{\left(1+\frac{\Delta v}{V_0}\right)}^{1.5}-{\lambda}_0^{-18}{\left(1+\frac{\Delta v}{V_0}\right)}^{-6}\right\}\right] $$

**Figure 4:**
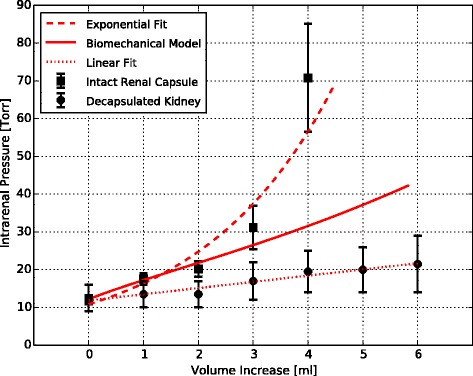
**Comparison between the biomechanical model of the renal capsule, the capsulated and decapsulated kidneys.** Data is expressed as mean value ± SEM.

The thickness of excised renal capsule has been previously reported [9], from which we set *H* = 100 μm. The parameters *R* = 6 × 10^5^ μm, *V*_0_ = 8 mL, and *λ*_0_ = 1.07 were determined from a curve fit to the experimental results, as shown in Figure 4. It is important to remark that the value of the stretch ratio *λ*_0_ is within the range of residual strain found in many biological tissues [12].

## Discussion

**Figure 5:**
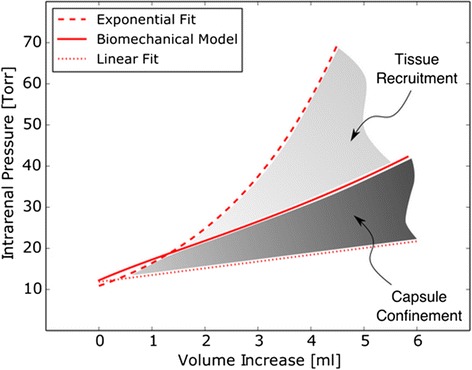
**Comparative plot of pressure-volume curves for the intact kidney, the decapsultaed kidney, and the biomechanical model.** The capsule confinement can explain the nonlinear relation between pressure and volume for small amounts of fluid. Tissue recruitment provides additional stiffness to the renal continent in order to bear the intrarenal overpressure.

A major result of this work is the nonlinear relation between intrarenal pressure and volume in the intact kidney, which is well described by an exponential relation. This finding is indicative of a mechanical behavior commonly observed in organs confined by a rigid or semi-rigid continent, like the cranial and intra-abdominal compartments. As a proof of concept, the pressure-volume curve protocol was performed on two kidneys previously decapsulated. The resulting pressure-volume curve is well explained by a linear relation (*R*^2^ = 0.95), in contrast to nonlinear dependence found in intact kidneys. The proposed biomechanical model was able to explain the increase in intrarenal pressure for volume increments up to 3mL in the experiments performed. However, for higher increments of volume, there is an abrupt increase in pressure that cannot be explained by the mechanical confinement provided by the renal capsule alone. From a purely mechanical point of view, an abrupt increase of the pressure, and therefore of the membrane tension, can be explained by an increase of the membrane thickness supporting the applied pressure. This can be inferred from Equation 1, by noting that, for a given volume, stress *σ* and radius *r* are fixed, and thus the only way to increase the intrarenal pressure *p* is by increasing the continent thickness *h*. Since the capsule thickness cannot grow spontaneously and, in fact can only shrink as the capsule stress increases, an adjacent layer of parenchymal tissue must carry the additional overpressure, a phenomenon we term here as tissue recruitment (Figure 5).

In AKI, a frequent finding is the increase in the kidney volume due to edema [4]. Further, hypoperfusion of the outer medulla is common in many forms of AKI [13-15]. In view of our results, hypoperfusion may be explained by a reduction of the renal perfusion pressure ostensibly caused by the increase of intrarenal pressure due to the volume increment. This idea is supported by the fact that the use of vasodilators to revert renal hypoperfusion has been ineffective to restore blood flow [14-18], indicating that renal perfusion is not controlled by vasomotor tone but rather by renal parenchymal pressure. Concordantly, recent studies in an ischemia-reperfusion murine model demonstrate that preventing the rise in intrarenal pressure caused by interstitial edema by making a small incision in renal capsule attenuates the risk of functional renal impairment. These findings suggest that a rise in parenchymal pressure may be contributed to the acute kidney injury caused by ischemic insult [7].

## Conclusions

We have studied the dependence of the intrarenal pressure on the fluid volume in the porcine intact kidney. A highly nonlinear relation between the intrarenal pressure and the injected volume was found, which confirms the existence of a mechanical behavior commonly observed in organs confined by a rigid or semi-rigid continent, which we refer to here as the renal compartment. In contrast, decapsulted kidneys present a pressure-volume linear relation, thus confirming the role of the renal capsule as a continent. From a biomechanical analysis, it can be concluded that the observed nonlinear pressure-volume relation cannot be solely explained by the confinement conferred by the renal capsule, suggesting that above a certain level of intrarenal pressure, tissue recruitment at the kidney periphery occurs in order to sustain higher levels of intrarenal pressure. The mechanical role of the renal capsule investigated in this work may have important implications in elucidating the role of decompressive capsulotomy in preventing the rapid intrarenal pressure increase in acutely injured kidneys (e.g., kidney transplantation). Future studies could assess the effect of renal decapsulation on renal blood flow, renal oxygenation and perfusion, microcirculation, and renal function in acutely injured kidneys.

## Abbreviations

AKI: acute kidney injury
RR: respiratory rate
PaCO_2_: partial pressure of carbon dioxide
PaO_2_: partial pressure of oxygen
